# From Neurodevelopmental to Neurodegenerative Disorders: The Vascular Continuum

**DOI:** 10.3389/fnagi.2021.749026

**Published:** 2021-10-20

**Authors:** Julie Ouellette, Baptiste Lacoste

**Affiliations:** ^1^Ottawa Hospital Research Institute, Neuroscience Program, Ottawa, ON, Canada; ^2^Department of Cellular and Molecular Medicine, Faculty of Medicine, University of Ottawa, Ottawa, ON, Canada; ^3^University of Ottawa Brain and Mind Research Institute, Ottawa, ON, Canada

**Keywords:** cerebrovascular abnormalities, neurodevelopment and intellectual disabilities, aging, neurodegeneration, cerebral blood flow, angiogenesis, blood-brain barrier

## Abstract

Structural and functional integrity of the cerebral vasculature ensures proper brain development and function, as well as healthy aging. The inability of the brain to store energy makes it exceptionally dependent on an adequate supply of oxygen and nutrients from the blood stream for matching colossal demands of neural and glial cells. Key vascular features including a dense vasculature, a tightly controlled environment, and the regulation of cerebral blood flow (CBF) all take part in brain health throughout life. As such, healthy brain development and aging are both ensured by the anatomical and functional interaction between the vascular and nervous systems that are established during brain development and maintained throughout the lifespan. During critical periods of brain development, vascular networks remodel until they can actively respond to increases in neural activity through neurovascular coupling, which makes the brain particularly vulnerable to neurovascular alterations. The brain vasculature has been strongly associated with the onset and/or progression of conditions associated with aging, and more recently with neurodevelopmental disorders. Our understanding of cerebrovascular contributions to neurological disorders is rapidly evolving, and increasing evidence shows that deficits in angiogenesis, CBF and the blood-brain barrier (BBB) are causally linked to cognitive impairment. Moreover, it is of utmost curiosity that although neurodevelopmental and neurodegenerative disorders express different clinical features at different stages of life, they share similar vascular abnormalities. In this review, we present an overview of vascular dysfunctions associated with neurodevelopmental (autism spectrum disorders, schizophrenia, Down Syndrome) and neurodegenerative (multiple sclerosis, Huntington’s, Parkinson’s, and Alzheimer’s diseases) disorders, with a focus on impairments in angiogenesis, CBF and the BBB. Finally, we discuss the impact of early vascular impairments on the expression of neurodegenerative diseases.

## Introduction

**FIGURE 1:**
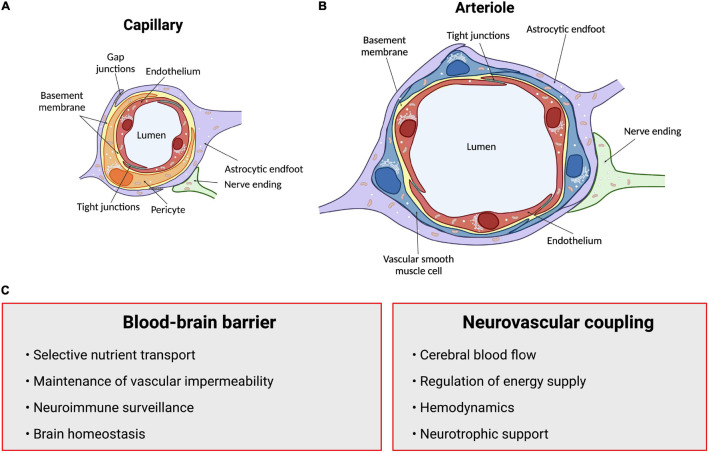
Summary of structures and functions of the neurovascular unit (NVU). The anatomical substrate for regulation of cerebral blood flow and the blood-brain barrier is a multicellular system consisting of neurons, pericytes, smooth muscle cells, astrocytes and endothelial cells known as the NVU. **(A)** Intracerebral capillaries lack vascular smooth muscle cells but are partly covered by contractile pericytes. **(B)** At the level of intracerebral arterioles, the endothelium is fully covered by a single layer of vascular smooth muscle cells, which provide contractile properties to the arteriole. Astrocytes send their processes called endfeet around both capillaries and arterioles, providing support as well as a functional connection to surrounding parenchyma. **(C)** Summary of functions of the blood-brain barrier and neurovascular coupling. Arrows point to neurovascular unit components. *Figure made using BioRender*.

The human brain contains approximately 100 billion vessels (∼600 km), all of which are critical for the delivery of nutrients and oxygen to neural cells (Quaegebeur et al., 2011). Although the brain accounts for only 2% of the body’s mass, it consumes about a quarter of the body energy produced at rest (Attwell et al., 2010). This colossal energy consumption is elemental to maintain normal functioning of the brain. Such energy requirements make the brain heavily reliant on key vascular features: (i) a dense vasculature to sustain adequate perfusion, (ii) a functional blood-brain barrier (BBB) to maintain brain homeostasis, and (iii) the proper regulation of cerebral blood flow (CBF) to match metabolic demands (Figure 1). Thus, a healthy brain vasculature is essential to support neural cells and ensure normal brain maturation, function and aging (Attwell and Laughlin, 2001; Girouard and Iadecola, 2006; Andreone et al., 2015; Lacoste and Gu, 2015). This is accomplished in part via neurovascular coupling (NVC) mechanisms that regulate CBF to support energetic demands of brain cells (Hamel, 2006; Attwell et al., 2010; Kaplan et al., 2020). While most studies are describing neurovascular signaling at the level of the microvasculature, other vascular segments have received very little attention. There is evidence suggesting that different vascular segments play different roles during vascular responses which is involved in maintaining brain homeostasis. The concept of heterogeneous vascular modules has been extensively reviewed in Schaeffer and Iadecola (2021).

**FIGURE 2:**
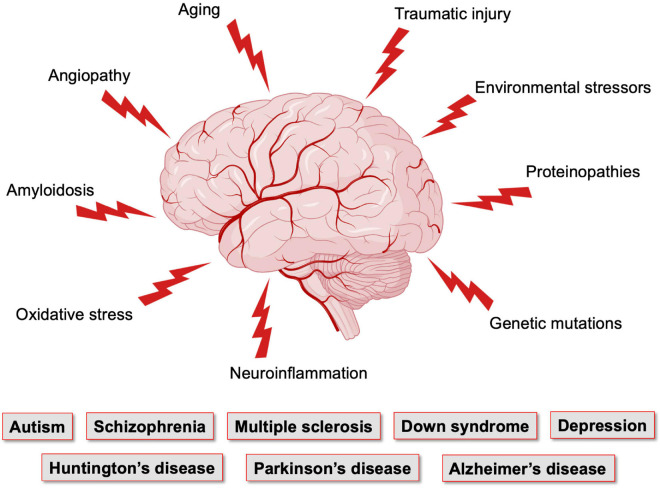
Factors affecting the brain vasculature and leading to neurological conditions. The brain, which has elevated metabolic needs but poor energy storage, is highly dependent on a continuous supply of nutrients and oxygen from the blood stream, and is thus dependent on the integrity of its vasculature. Vasculature of the brain is the most complex and dense in the human body. Yet, it is maintaining a very fragile equilibrium and is the target of numerous pathological conditions that affect neuronal maturation and function. *Figure made using BioRender*.

The close anatomical apposition between the nervous and vascular systems supports a functionally integrated network (Attwell et al., 2010; Lecrux and Hamel, 2011; Hillman, 2014; Huneau et al., 2015; Kaplan et al., 2020). This involves modulating vascular tone by secretion of vasoconstrictor and vasodilator molecules. Initially, it was proposed that local metabolic factors released by neurons modulate local CBF (Sherrington, 1890; Friedland and Iadecola, 1991). Since then, several studies have introduced other cellular mediators of NVC which altogether form the neurovascular unit (NVU). This anatomical substrate of NVC indeed involves a multicellular system consisting of neurons, pericytes, smooth muscle cells, astrocytes, microglia and endothelial cells (ECs) that together orchestrate CBF, and thus brain function (Attwell et al., 2010; Andreone et al., 2015; Grubb et al., 2021; Figure 1). The cerebral cortex is innervated by projection neurons that release neurotransmitters including, but not limited to, acetylcholine, noradrenaline, serotonin and glutamate, involved in the regulation of vessel diameter (Sandoo et al., 2010). Pericytes, while having debated roles in NVC, possess contractile properties and regulate blood flow around capillaries (Attwell et al., 2010, 2016; Fernandez-Klett and Priller, 2015; Sweeney et al., 2018; Watson et al., 2020; Hartmann et al., 2021). Capillary pericytes are α-smooth muscle actin (SMA)-negative and only partially cover the vessel, while ensheathing pericytes are α-SMA-positive, occupy proximal branches of penetrating arteriole offshoots, and fully cover the vessels. However, they are classified as different from smooth muscle cells as they display an ovoid cell body (Grant et al., 2019). Vascular smooth muscle cells (SMCs), found on intracerebral arterioles and arteries, are absent from intracerebral capillaries. These cells are short, densely packed, ring-shaped, and essential for regulating vessel tone (Lacoste and Gu, 2015; Frosen and Joutel, 2018; Grant et al., 2019). Astrocytes occupy a critical position between blood vessels and neurons. They can modulate vessel tone via receptor-mediated increase in astrocytic Ca^2+^, resulting in the release of astrocyte-derived prostaglandins (PGE_2_), nitric oxide (NO), epoxyeicosatrienoic acids (EETs), glutamate, or adenosine, all of which can alter vascular diameter and tone (Attwell et al., 2010; Cauli and Hamel, 2010; Filosa and Iddings, 2013; Harada et al., 2015; Haidey et al., 2021), as reviewed in detailed elsewhere (Filosa and Iddings, 2013; Howarth, 2014; MacVicar and Newman, 2015; Mishra, 2017; McConnell et al., 2019; Stackhouse and Mishra, 2021). Whereas microglia are the main regulators of inflammatory processes in the brain, their role in NVC is not well defined. However, recently, they were suggested as essential in regulating CBF during neural activation (Császár et al., 2021). Brain ECs have unique morphological and functional features such as a lack of fenestration, the presence of tight junctions between cells, a low number of pinocytic vesicles that limit transcytosis, hence forming the first limiting layer of the BBB (Reese and Karnovsky, 1967; Stamatovic et al., 2008; Rizzo and Leaver, 2010; Salmina et al., 2014; Andreone et al., 2015). This highly selective barrier promotes a tightly regulated brain homeostasis to ensure proper neuronal function, protecting the brain from toxins, pathogens, inflammation, and injury (Weiss et al., 2009; Larsen et al., 2014; Daneman and Prat, 2015; Van Dyken and Lacoste, 2018). Furthermore, brain ECs regulate vascular tone by releasing vasodilators including endothelial-derived NO, endothelium-derived EETs, PGE_2_ and prostacyclin, as well as vasoconstrictors such as endothelin-1, thromboxane A_2_ and prostaglandin F_2__α_ (Mohan et al., 2012; Filosa and Iddings, 2013; Andreone et al., 2015; Kisler et al., 2016, 2017; Dabertrand et al., 2021). While the endothelium regulates vascular permeability and tone, it is also the main target of small vessel disease (SVD), which refers to a pathological process that damages arterioles, venules and brain capillaries. SVD has a major impact on CBF and cognition (Hakim, 2019). The NVU as a whole is also responsible for maintaining BBB integrity (Abbott et al., 2006; Zlokovic, 2008; Daneman, 2012; Kadry et al., 2020). Alterations in vascular patterning, CBF and BBB, either during development or later in life, contribute to the onset and/or progression of early- or late-onset neurological disorders (Figure 2).

Well-balanced vascular and neuronal interactions are required to support brain function from early life. The shared spatial and temporal patterns of vascular and neuronal networks suggest an integrative role for vessels in neural development, and *vice versa* (Gu et al., 2005; Carmeliet and Jain, 2011; Andreone et al., 2015; Lacoste and Gu, 2015). Neurovascular crosstalk, which initially takes place during embryogenesis, supports the rising oxygen and nutrient demand of immature neurons as they require extensive energy to maintain normal course of development (De Filippis and Delia, 2011). The increased energy consumption by neurons creates a hypoxic environment acting as a signal for boosting blood vessel production to upsurge delivery of oxygen and metabolic substrates to the brain (Stone et al., 1995; Lacoste and Gu, 2015; Peguera et al., 2021). Hypoxia initiates vessel ingression into deep brain structures, followed by usage of vascular patterning cues (Lacoste and Gu, 2015; Tata et al., 2015; Tata and Ruhrberg, 2018; Okabe et al., 2020). Comparably, ECs instruct neural progenitors into dividing, differentiating or migrating through release of paracrine signals that regulate neuronal development in vascular niches (Hogan et al., 2004; Shen et al., 2004; Daneman et al., 2009; Goldman and Chen, 2011; Delgado et al., 2014; Lacoste and Gu, 2015; Licht and Keshet, 2015; Walchli et al., 2015; Tata and Ruhrberg, 2018; Peguera et al., 2021). Moreover, neuronal activity plays important roles in modulating postnatal brain angiogenesis (Lacoste et al., 2014; Whiteus et al., 2014; Biswas et al., 2020). As the brain matures, vascular networks remodel until the system consists of an extensive network that actively regulates blood flow to adequately sustain energy demands. The functional relationships between neurons and blood vessels ensures that NVC mechanisms progressively develop (Lacoste and Gu, 2015; Coelho-Santos and Shih, 2020). NVC becomes fully functional ∼3–4 weeks after birth in rodents, and 7–8 weeks in humans (Yamada et al., 2000; Muramuto et al., 2002; Kozberg et al., 2016).

**FIGURE 3:**
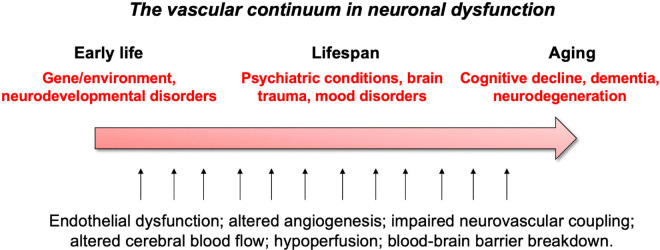
Summary of vascular links to neurological disease throughout life.

These vascular features can become defective early in life, affecting brain maturation. Vascular susceptibilities can also emerge later in life, taking part in neurodegenerative processes. Indeed, NVU deficits play a role in both early- and late-onset neurological disorders (Figure 2). Mounting evidence shows that vascular impairments contribute to the pathophysiology of neurological conditions throughout life, including neurodevelopmental, metabolic, and neurodegenerative disorders (Nicolakakis and Hamel, 2011; Van Dyken and Lacoste, 2018; McConnell et al., 2019; Ouellette et al., 2020; Sharma and Brown, 2021). This suggests the existence of a vascular continuum between developmental conditions and illnesses of aging, which will be the focus of this review (Figure 3). A better understanding of mechanisms and key players involved in cerebrovascular impairments may lead to transformative therapeutic strategies at different stages of life.

## Cerebrovascular Deficits Associated With Neurodevelopmental Disorders

Neurodevelopmental disorders are considered a group of conditions with onset/diagnosis during infancy, childhood, or adolescence (Morris-Rosendahl and Crocq, 2020). They are defined by impairments in motor, social, cognitive, academic, and/or occupational functioning. Most studies focused on the neuronal contributions to these disorders; however, concomitant vascular impairments are starting to emerge (Ouellette et al., 2020). Here, we highlight vascular impairments identified in autism spectrum disorders (ASD) and schizophrenia.

### Vascular Links to Autism Spectrum Disorders

ASD are pervasive neurodevelopmental disorders associated with social interaction deficits, speech and language impairments, as well as repetitive behaviors and restricted interests (Vijayakumar and Judy, 2016). These disorders have a prevalence of 1–2% in the general population and affect four times more boys than girls (Hogan et al., 2004; Daneman et al., 2009). Individuals with ASD show atypical behaviors associated with visual attention, imitation, social responses, and motor control by 12 months of age. By the age of 3, a child can be efficiently diagnosed with ASD (Park et al., 2016). While the underlying causes of ASD are enigmatic, both environmental and genetic origins have been found, leading to the identification of gene mutations within the ASD population (Hogan et al., 2004; James et al., 2009; Emerson et al., 2017). Although most studies have been neurocentric, ASD are now being associated with vascular vulnerabilities.

#### Altered Cerebral Blood Flow in Autism Spectrum Disorders

Neuroimaging techniques can map changes in CBF or blood oxygenation during various activities. Morphological and functional investigations using functional magnetic resonance imaging (fMRI), positron emission tomography (PET), single-photon emission computed tomography (SPECT), or Arterial Spin Labeling (ASL) are used to measure CBF changes in ASD children. CBF disruptions have been demonstrated in ASD patients when compared to healthy controls in different regions of the brain (Bjørklund et al., 2018). It has also been suggested that perfusion alterations are more pronounced in older children diagnosed with ASD. Cerebral hypoperfusion has been detected in nearly 75% of ASD children (Zilbovicius et al., 2000). As CBF impacts the delivery of oxygen and nutrients to neurons, hypoperfusion in ASD children has been associated with key ASD-related behaviors such as language deficits, impaired executive function and abnormal responses to sensory stimuli, as well as difficulty in facial perception (Siegel et al., 1992; Chiron et al., 1995; Ohnishi et al., 2000; Burroni et al., 2008; Reynell and Harris, 2013; Bjørklund et al., 2018; Yerys et al., 2018). These behaviors correlate with abnormal regional cerebral blood flow (rCBF) in the bilateral insula, superior temporal gyri and left prefrontal cortices, medial temporal lobe, supramarginal gyrus, right fusiform gyrus, and dorsal anterior cingulate cortex (Ohnishi et al., 2000; Zilbovicius et al., 2000; Burroni et al., 2008; Jann et al., 2015; Yerys et al., 2018). Studies are attempting to ameliorate these behavioral abnormalities using hyperbaric oxygen treatment (HBOT) to counteract cerebral hypoperfusion in children with ASD. There is some evidence that children who undertook 40 HBOT sessions of 60 min each showed improvements on selected psychosomatic parameters in the Autism Treatment Evaluation Checklist (ATEC) and Childhood Autism Rating Scale (CARS) (Kostiukow and Samborski, 2020). Currently, there is insufficient evidence to support the use of HBOT to treat children with ASD as there are many contradicting studies claiming no improvement in behaviors. Nevertheless, each study followed different protocols, consisted of patients with a large spectrum of behavioral impairments, and some lacked proper control groups, which could explain discrepancies. More research is required to determine if specific groups of children could benefit from HBOT treatment (Rossignol et al., 2012; Sakulchit et al., 2017).

ASL-based measurements of cerebral perfusion showed that children with ASD presented a pattern of widespread hyperperfusion in frontotemporal regions including medial orbitofrontal cortex, bilateral inferior frontal operculum, left inferior/middle temporal gyrus and the right precentral gyrus (Jann et al., 2015). The medial orbitofrontal cortex is known to have extensive connections with the limbic system involved in socio-emotional cognition. Furthermore, hyperperfusion was detected throughout the frontal white matter and subcortical gray matter in ASD children, which correlated positively with severity of social deficits (Peterson et al., 2019). As shown by these studies, CBF abnormalities appear linked to clinical manifestations. Although opposing observations of CBF in ASD patients were reported, these further support the complexity of these disorders (Jann et al., 2015).

Neurovascular coupling alterations were also observed in ASD patients. Hemodynamic responses in children with ASD during a color-word task were significantly lower than the control group, especially in the dorsolateral prefrontal cortex (Uratani et al., 2019). Conversely, children displayed no difference in hemoglobin concentrations in the prefrontal cortex during a letter fluency task, while adults showed reduced responses (Kawakubo et al., 2009). Despite inter-study variability, there seems to be a consensus on the impact of altered CBF on the expression of behavioral impairments (Zilbovicius et al., 2000; Jann et al., 2015). But as ASD are heterogeneous, with various behavioral traits, genetic causes, medical co-morbidities and medications, these variables may have impacted neuroimaging results, which led to inconsistencies. Importantly, these studies take an important step toward the identification of key players in ASD pathophysiology, opening new opportunities for early diagnosis and treatment.

The relationship between CBF alterations and symptom profiles in ASD children provides insight into disease mechanisms that can be tested in animal models. As most pre-clinical studies have also focused on the neuronal aspects of ASD, very few have considered vascular contributions to these disorders in laboratory models. Recent studies using different ASD mouse models have reported alteration in CBF. A study by Abookasis et al. (2018) using inbred Black and Tan Brachyury (BTBR) *T+tf/J* reported decreased CBF in mutant mice using laser speckle imaging and laser Doppler flowmetry (LDF). Subsequently, work by Ouellette et al. (2020) using the 16p11.2 deletion mouse model of ASD (*16p11.2*^*df/*+^; Horev et al., 2011) demonstrated an increase in resting CBF as well as neurovascular uncoupling in adult (P50) *16p11.2*^*df/*+^ mice compared to WT littermates using a combination of ultrasound imaging and LDF. No difference in CBF or NVC were observed between younger (P14) mutant and control mice (Ouellette et al., 2020). Results from this study in *16p11.2*^*df/*+^ mice revealed the cause of these functional cerebrovascular impairments: an endothelial deficit. While normal vascular smooth muscle cell function was measured, defective endothelium-dependent vasodilation was found *ex vivo* following exposure to specific vasomodulators (Ouellette et al., 2020). This suggests that endothelial health plays an important role in the etiology of the 16p11.2 deletion ASD syndrome. Understanding the molecular and cellular factors that mediate CBF alterations in ASD could help design rescue approaches in animal models, as well as therapeutic approaches down the line.

Since MRI studies rely on Blood Oxygen Level Dependent (BOLD) signals as surrogates for neuronal activity (Hillman, 2014; Howarth et al., 2021; Moon et al., 2021), it is possible that changes in rCBF reflect changes in underlying neuronal activity. For instance, cerebral cortex hypoperfusion in ASD patients could reflect lower metabolic demands (Schifter et al., 1994). In the case *16p11.2*^*df/*+^ mice, however, it is interesting to note that a neurovascular uncoupling was measured, with enhanced neuronal activation yet reduced vascular responses to whisker stimulations, which led to the discovery of endothelium-dependent deficits (Ouellette et al., 2020).

#### Altered Blood-Brain Barrier and Angiogenesis in Autism Spectrum Disorders

Cerebral vessels are central for the maintenance of brain homeostasis, sustaining proper neuronal function, and providing an effective protection against toxins and pathogens (Profaci et al., 2020). The BBB consists of specialized ECs lining the vessel wall to separate the peripheral blood from cerebral tissue. Brain (central) ECs are distinct from peripheral ECs, as they produce specific proteins to control the flux (entry and exit) of metabolites across vessels, to maintain low rates of trans-endothelial vesicular transport, and to form tight junctions to limit the para-cellular flow of material between adjacent ECs (Andreone et al., 2015; Chow and Gu, 2015; Kealy et al., 2020). Alterations in the BBB are at the core of the onset and/or progression of numerous neurological disorders (Daneman and Prat, 2015; Van Dyken and Lacoste, 2018; Profaci et al., 2020). Only few studies have investigated the components of the BBB in the context of ASD. Children diagnosed with ASD have been associated with reduced levels of adhesion molecules such as soluble Platelet Endothelial Cell Adhesion Molecule-1 (PECAM-1, or CD31) and P-selectin. Since these molecules are essential to modulate BBB permeability through signaling and leukocyte infiltration, it suggests that crucial BBB components may be at play in ASD pathophysiology (Onore et al., 2012). Furthermore, a post-mortem study, with a small sample size, demonstrated altered BBB integrity in ASD with increased gene expression of matrix metalloproteinase (MMP)-9 (Fiorentino et al., 2016). Studies have shown that MMP-9 regulates cell proliferation, adhesion, degradation of laminin and collagen, angiogenesis, oxidative injury, and is implicated in BBB breakdown (Lepeta and Kaczmarek, 2015; Turner and Sharp, 2016). Additionally, important components of BBB integrity displayed altered expression in ASD patients, including claudin-5 (*CLDN5*) and claudin-12 (*CLDN12*), as well as tricellulin (*MARVD2*), a component of tight junctions involved in decreased permeability to macromolecules in brain ECs (Fiorentino et al., 2016). In an older study, a small subset of ASD participants demonstrated higher levels of autoantibodies against brain ECs in the serum compared to typically developing individuals, suggesting an impact on the BBB (Connolly et al., 1999). Animal models have facilitated the study of BBB integrity in ASD. In a valproic acid rat model of autism, increased BBB permeability to Evans blue was found in the cerebellum, a phenotype attenuated by treatment with memantine, an NMDA receptor modulator. This BBB alteration was also attenuated using minocycline (antibiotic) and agomelatine (melatonin receptor) treatment (Kumar et al., 2015; Kumar and Sharma, 2016). Animal studies have investigated transendothelial transport mechanisms in ASD mouse models. Tarlungeanu et al. (2016) demonstrated that the large neutral amino acid transporter (LAT1, *Slc7a5*) localized at the BBB to maintain normal levels of brain branched chain amino acid (BCAA) was required for neurotypical development. Mice harboring an endothelial-specific deletion of *Slc7a5* (*Slc7a5^Δ*EC*^*) displayed behaviors reminiscent of ASD, including motor dysfunctions consistent with a study in human patients harboring the constitutive mutation (Novarino et al., 2012; Tarlungeanu et al., 2016). Interestingly, administration of BCAA rescued ASD-like behaviors in *Slc7a5^Δ*EC*^* mice (Tarlungeanu et al., 2016).

Recently, a post-mortem analysis of brain tissue from individuals diagnosed with ASD revealed significantly higher levels of markers associated with pericytes, as well as increased vascular tortuosity, indirectly suggesting impairments in angiogenesis, a process through which new blood vessels are formed (Azmitia et al., 2016). A more recent study in *16p11.2*^*df/*+^ mice revealed impaired cerebral angiogenesis in young (P14) *16p11.2*^*df/*+^ male mice compared to sex-/age-matched littermates, a phenotype which was absent in adult mice. Defective angiogenic activity was also measured using primary brain ECs from P14 *16p11.2*^*df/*+^ males or ECs derived from human-induced pluripotent stem cells (hiPSCs) of 16p11.2 deletion carriers (Ouellette et al., 2020). Moreover, RNA-sequencing analysis of *16p11.2*^*df/*+^ mouse brain EC transcriptome revealed changes in the expression of genes involved in angiogenesis (e.g., *Grem1*, *Apln*, *Angpt2*), while key genes involved in BBB regulation (e.g., *Pecam1*, *Mfsd2a*, *Cldn5*, *Slc2a1*) were not affected by the 16p11.2 deletion (Ouellette et al., 2020). Finally, this study generated a mouse model with endothelial-specific 16p11.2 haploinsufficiency which recapitulated ASD-related phenotypes, revealing a causal relationship between endothelial dysfunction and neuronal aspects of the 16p11.2 deletion syndrome (Ouellette et al., 2020).

Overall, these studies allude to the contribution (structural and functional) of a defective BBB and NVU in ASD, with an important role for endothelial impairments.

### Vascular Links to Schizophrenia

Schizophrenia is a debilitating neurodevelopmental disorder affecting ∼1% of the population. It is associated with behavioral and cognitive symptoms that arise progressively. Memory and attention deficits appear in childhood, while positive symptoms (psychotic episodes) and negative symptoms (social and motivational deficits) emerge later in adolescence or early adulthood (Stachowiak et al., 2013). Although the incidence of schizophrenia is higher in men, women have a slightly later disease onset (Gogtay et al., 2011; Ochoa et al., 2012). While the behavioral aspects of schizophrenia have been described, the causes of this disorder are poorly known. As in ASD, both genetic and environmental origins are involved. Schizophrenia has been associated with genes essential for a wide range of functions including neuronal connectivity and patterning of brain structures, cell proliferation and differentiation, as well as cytoskeleton reorganization (Stachowiak et al., 2013; Clifton et al., 2019). As in most neurological disorders, the implication of neuronal alterations has been extensively studied, but research on vascular impairments in schizophrenia is starting to emerge.

#### Altered Cerebral Blood Flow in Schizophrenia

Cognitive impairments are often present before the first psychotic episode in patients with schizophrenia (Keefe and Harvey, 2012; Schuepbach et al., 2016) and deficits in executive functions are often parallel to changes in CBF. Several studies have linked altered CBF with schizophrenia-related symptoms (Sabri et al., 1997; Malaspina et al., 1999, 2004; Pinkham et al., 2011; Fujiki et al., 2013; Schuepbach et al., 2016; Stegmayer et al., 2017; Zhu et al., 2017). Interestingly, the manifestations of negative or positive symptoms correlate with different rCBF changes. In a study by Pinkham et al. (2011), CBF of 30 schizophrenia patients was measured using ASL perfusion MRI, which revealed a positive correlation between increased severity of positive symptoms and higher CBF in the cingulate and superior frontal gyri, but decreased CBF in precentral and middle frontal gyri. Patients who presented with severe negative symptoms also displayed reduced CBF in the superior temporal gyrus bilaterally, cingulate and left middle frontal gyri (Malaspina et al., 2004; Scheef et al., 2010; Pinkham et al., 2011; Liu et al., 2012). Most studies investigating CBF alterations in schizophrenia considered perfusion rates from medicated patients, and a small number of studies have measured CBF rates in neuroleptic-naïve patients. Using ASL in non-medicated patients, the schizophrenia group displayed resting-state hypoperfusion in the frontal lobes, anterior and medial cingulate gyri, as well as in the parietal lobes, while increased perfusion was measured in the cerebellum, brainstem and thalamus (Scheef et al., 2010). Sabri et al. (1997) measured rCBF using SPECT in non-medicated patients that have experienced positive symptoms, revealing that rCBF values varied depending on the severity of positive symptoms. Hyperperfusion was detected in the frontal, anterior cingulate as well as in both parietal and temporal cortices in patients who had scored high in severity for formal thought disorder (disturbance of the organization and expression of thought). In contrast, patients who scored high for delusions, hallucinations or distrust, with low scores for formal thought, displayed hypoperfusion in the same brain regions. No difference in rCBF was identified between control and schizophrenia groups after treatment (Andreasen et al., 1997; Sabri et al., 1997; Horn et al., 2009). Recent studies have detected hyperperfusion and hypoperfusion in brain regions from individuals with hallucinations. For instance, CBF increase was found in the right superior temporal gyrus and caudate nucleus, while CBF decrease was found bilaterally in the occipital and left parietal cortices (Zhuo et al., 2017). In another study, patients were classified based on the severity of three behavioral dimensions (language, affectivity, and motor) according to the Bern Psychopathology scale. Patients with altered affectivity were associated with increased CBF in the amygdala, while changes in language dimension were linked to increased CBF in Heschl’s gyrus (Stegmayer et al., 2017). While schizophrenia is classified as a neurodevelopmental disorder, its symptoms persist with age. Studies have identified significant bilateral temporal hypoperfusion related to aging and disease course. It has been suggested that this decrease in CBF with aging is paralleled with the degenerative changes observed in patients with schizophrenia (Schultz et al., 2002; Kawakami et al., 2014).

The polygenic risk of schizophrenia is an important dimension of this syndrome, and changes in CBF have been identified in patients diagnosed for either familial or sporadic schizophrenia. Sporadic schizophrenia patients were associated with hypofrontality (left frontal gyrus, orbitofrontal cortex, anterior cingulate, and paracingulate cortices), while familial schizophrenia patients had left temporoparietal hypoperfusion (posterior Sylvian fissure at the superior and inferior parietal lobules, angular, and supramarginal gyri). In both groups, positive symptoms are often associated with increased rCBF in the parahippocampal gyrus, cerebellum, and pons (Malaspina et al., 2004). Sporadic patients showed additional hyperperfusion in the fusiform gyrus, and familial patients the hippocampus, dentate, amygdala, thalamus, and putamen (Malaspina et al., 2004). In addition, the prefrontal cortex in schizophrenia has been associated with deficits of pericapillary oligodendrocytes, which could contribute to changes in CBF (Vostrikov et al., 2008; Uranova et al., 2010). Altogether, these studies support the idea that altered CBF is involved in schizophrenia pathophysiology.

In addition to studies investigating resting state CBF, there is evidence of altered NVC in schizophrenia whereby many reports demonstrate reduced hemodynamic response, reflecting reduced neuronal activity during processing of cognitive tasks, especially in the lateral prefrontal cortex and temporal regions (Ford et al., 1999, 2005; Mathalon et al., 2000; Carter et al., 2001; Hanlon et al., 2016; Pu et al., 2016). As with CBF reports, there are inconsistent hemodynamic responses associated with schizophrenia since increased hemodynamic responses in hippocampus, thalamus and prefrontal cortex have been identified (Tregellas et al., 2007). These conflicting results are translating to rodent models of schizophrenia whereby some models have revealed overall hypofrontality, hypoperfusion in the hippocampus or hyperperfusion in the somatosensory cortex (Finnerty et al., 2013; Song et al., 2013; Drazanova et al., 2019).

Altogether, these studies support the idea that altered CBF regulation is involved in schizophrenia pathophysiology. Moreover, it appears critical to consider the polygenic risk of disease, the category and severity of symptoms, as well as the age of patients when comparing CBF rates in schizophrenia. Although many studies have detected altered CBF using various methods, results thus far remain conflicting based on various stages of disease and pharmacological treatment (Drazanova et al., 2019).

#### Altered Blood-Brain Barrier and Angiogenesis in Schizophrenia

A dysfunctional BBB has been reported in schizophrenia, with increased permeability to damaging proteins (Müller and Ackenheil, 1995; Shcherbakova et al., 1999; Crockett et al., 2021). Studies are starting to decipher changes in cells associated with the BBB (for a detailed review, see Carrier et al., 2020). Briefly, evidence of schizophrenia-associated microvascular abnormalities in the neocortex include thickening and deformation of basal lamina, vacuolation of cytoplasm in ECs, basal lamina and astrocytic end-feet, swelling of astrocyte end-feet, activation of microglial cells in the prefrontal and visual cortex, as well as atypical vascular arborization (Uranova et al., 2010; Carrier et al., 2020).

Moreover, specific mutations are associated with schizophrenia, including alterations in the 22q11.2 deletion syndrome (22qDS) -strongest monogenic risk allele for this disorder, and polymorphisms in claudin-5, a densely expressed tight junction molecule (Gur et al., 2017; Greene et al., 2018; Carrier et al., 2020) altogether revealing barrier dysfunction in schizophrenia patients (Greene et al., 2018; Crockett et al., 2021). Post-mortem brain sections from 22qDS patients and animal models of 22qDS both demonstrate reduced claudin-5 expression in the BBB, which in turn compromised BBB function (Nishiura et al., 2017; Guo et al., 2020; Crockett et al., 2021; Usta et al., 2021). Additionally, altered levels of vascular endothelial (VE)-cadherin and occludin in ECs were identified in schizophrenia. These molecules regulate adherence of ECs and restrict movement of substances across the BBB (Cai et al., 2020). Furthermore, BBB hyperpermeability has been associated with another risk allele for schizophrenia. *NDST3*, expressed in the brain, encodes an enzyme involved in the metabolism of heparan sulfate, a component of basal lamina extracellular matrix that is required for BBB integrity (Khandaker et al., 2015).

Studies have documented primary vascular endothelial dysfunction in schizophrenia. Individuals carrying *MTHER T* and/or *COMT Val* risk allele have been associated with cerebrovascular endotheliopathy, as well as lower frontal executive functions (Grove et al., 2015). While endothelial dysfunction is possibly associated with schizophrenia, many studies are using peripheral endotheliopathy as a surrogate marker for endothelial dysfunction. For example, studies are using non-invasive peripheral arterial tonometry (RH-PAT) to assess peripheral arteriole endothelial-dependent vasodilation and revealed impaired peripheral arterial vasodilation in schizophrenia (Ellingrod et al., 2011; Burghardt et al., 2014). Notably, brain ECs have unique properties to maintain BBB integrity and brain homeostasis. Although altered endothelial function was found in the periphery, it does not represent a definite marker of brain (central) endothelial dysfunction. A critical regulator of angiogenesis, *vascular endothelial growth factor* (VEGF), and its receptor (VEGFR2) have been found upregulated in the prefrontal cortex of individuals diagnosed with schizophrenia (Hino et al., 2016). Findings of elevated VEGF could also be linked to vascular hyperpermeability, as VEGF not only regulates angiogenesis but increases BBB leakage (Mayhan, 1999; Zhang et al., 2000). Conversely, a different group revealed that a decreased production of VEGF predisposed individuals to develop this disorder and contributed to the severity of symptoms (Saoud et al., 2021). Another study investigated the impact of hiPSC-derived neural stem cells from schizophrenia patients on angiogenesis (Casas et al., 2018). This study found an imbalance in the expression and secretion of several angiogenic factors and non-canonical neuro-angiogenic guidance cues from neural stem cells from schizophrenic patients. Conditioned media from these cells induced impaired angiogenesis as evidenced by reduced number of sprouts and tubes formed in *in vivo* and *in vitro* models, as well as decreased neural stem cell migration compared to control conditioned media (Casas et al., 2018).

## Cerebrovascular Deficits Associated With Neurodegenerative Disorders

CNS disorders are dichotomized as early onset neurodevelopmental disorders and late-onset neurodegenerative diseases (Taoufik et al., 2018). Neurodegenerative diseases consist of a group of heterogeneous disorders characterized by the progressive degeneration of structure and function in the CNS (Gitler et al., 2017). Although neurodegenerative and neurodevelopmental disorders are differentially classified, an accumulating body of work demonstrates significant similarities between these two groups of conditions. Here below, we cover cerebrovascular impairments reported in four neurodegenerative diseases that emerge throughout lifespan: multiple sclerosis (MS), Huntington’s disease (HD), Parkinson’s disease (PD), and Alzheimer’s disease (AD).

### Vascular Links to Multiple Sclerosis

MS is a chronic autoimmune disease of the CNS, occurring when the immune system attacks its own nerve fibers and myelin sheaths (D’Haeseleer et al., 2013). The pathological hallmark of MS consists of perivenular inflammatory lesions, leading to demyelinating plaques and diffuse axonal degeneration throughout the CNS (Dobson and Giovannoni, 2019). It is characterized by the infiltration of T cells reactive against myelin in the CNS (Schwartz and Kipnis, 2005). This demyelinating disease has key features including inflammation, BBB disruption and neurodegeneration. MS has a prevalence of 0.5–1.5 per 100,000 individuals, whereby women are three times more affected than men (Harbo et al., 2013). The age of MS onset is situated between 20 and 40 years of age (Ortiz et al., 2014). General symptoms related to MS include, but are not limited to, tremors, lack of coordination as well as weakness in limbs. There are various types of MS including relapsing-remitting MS (RR-MS), secondary progressive MS (SP-MS) and primary progressive MS (PP-MS). RR-MS consists of unpredictable relapses or inflammatory flare-ups during which new symptoms appear or existing ones worsen (Adhya et al., 2006). Most people with RR-MS, transition to a disease phase known as SP-MS. In this phase, there is progressive worsening and fewer relapses. Active lesions with profound lymphocytic inflammation are mostly found in RR-MS (Dobson and Giovannoni, 2019). PP-MS is considered as a slow accumulation of disability without defined relapses. In this case, PP-MS is associated with an inactive lesion core surrounded by activated microglia and macrophages (Dobson and Giovannoni, 2019).

#### Altered Cerebral Blood Flow in Multiple Sclerosis

MS has been associated with functional cerebrovascular abnormalities including decreased cerebral perfusion and reduced CNS venous blood drainage, known as chronic cerebrospinal venous insufficiency (D’Haeseleer et al., 2011). SPECT, PET, and ASL imaging studies have reported decreased CBF in both gray and white matter of MS patients (Ge et al., 2005; D’Haeseleer et al., 2011). Widespread cerebral hypoperfusion has been revealed in SP-MS, RR-MS and PP-MS patients, while an ischemic threshold was not reached (Adhya et al., 2006; Ota et al., 2013; Monti et al., 2018). Gray matter hypoperfusion in MS suggests a reduction of metabolism due to the loss of cortical neurons (Peruzzo et al., 2013). Furthermore, studies have reported that CBF is globally impaired in normal appearing white matter (NAWM) of patients with early RR-MS (Law et al., 2004; Adhya et al., 2006). Of note, CBF was generally lower in PP-MS than in RR-MS in the periventricular and frontal white matter (Adhya et al., 2006). In the contrary, other studies have measured elevation of CBF and cerebral blood volume (CBV) in NAWM of patients with early RR-MS several weeks before signs of increased BBB permeability (Wuerfel et al., 2004). Although studies on different types of MS revealed changes in CBF, general active demyelinating lesion regions are associated with hyperperfusion while the more stable forms show hypoperfusion (Monti et al., 2018). Decreased CBF in cerebral NAWM, thalamus, and putamen was identified in patients whose symptoms emerged within the first 5 years of onset. This suggests that CBF alterations are present in the very early stages of the disease (Varga et al., 2009). Different mechanisms have been proposed to explain hypoperfusion in MS. A study suggested that decreased CBF is secondary to axonal degeneration, which leads to a decreased metabolic demand (Saindane et al., 2007). However, this hypothesis is yet to receive experimental support. A second mechanism that has been proposed is an impaired energy metabolism of astrocytes (De Keyser et al., 1999). In MS, astrocytes are deficient in β2-adrenergic receptors which regulate high energy-consuming activities, such as glycogenolysis and phosphocreatine metabolism (De Keyser et al., 1999). Reduced energy production in astrocytes could be contributing to altered CBF. A third mechanism suggested was increased release of vasoconstrictor endothelin-1 (ET-1) from reactive astrocytes, found in a post-mortem study on white matter samples of RR-MS patients (D’Haeseleer et al., 2013; Hostenbach et al., 2019). Hence, elevated levels of ET-1 could be involved in dysregulating CBF in MS. Interestingly, administration of ET-1 antagonist Bosentan restored CBF to control levels in MS patients (D’Haeseleer et al., 2013).

Impaired cerebral vascular reactivity was evidenced in MS patients exposed to hypercapnia, which has been suggested to contribute to neuronal death identified in this disorder (Marshall et al., 2014). This global deficit is thought to be associated with elevated levels of NO reported in MS (Trapp and Stys, 2009; Juurlink, 2013). These studies suggest that the overproduction of NO may desensitize endothelial and smooth muscle cell function, causing decreased vasodilatory capacity and limited blood supply for neurons that perform demanding tasks. Increased NO in MS may lead to neuronal activity-induced hypoxia leading to neurodegeneration (Marshall et al., 2014). Interestingly, high inflammatory MS lesion load has been associated with increased CBF. Therefore, perfusion changes may be sensitive to active inflammation (Bester et al., 2015). However, it remains unclear whether abnormal perfusion in MS is a precursor of lesions or occurs independently of lesion development (Marshall et al., 2014).

Notably, MS has been associated with cerebral SVD. It was demonstrated that younger MS cases are more severely impacted by cerebral SVD compared to older individuals (Geraldes et al., 2020). This suggests that the interaction between MS and cerebral SVD is affected by age, an assumption still under investigation (Geraldes et al., 2020).

#### Altered Blood-Brain Barrier and Angiogenesis in Multiple Sclerosis

BBB dysfunction is considered a major hallmark of MS and is deemed a trigger of disease onset (McQuaid et al., 2009; Cramer et al., 2014). Intense focal disruption of the BBB associated with inflammation (identified by gadolinium-enhanced MRI at acute and chronic MS lesion sites; Saade et al., 2018) and diffuse extensive BBB disruption with a long-term pathological activity, are both found in MS patients (Bennett et al., 2010). Hyperpermeability of the BBB was evidenced by leukocyte passage across the BBB (Cramer et al., 2014). Increased BBB leakage was associated with decreased expression of tight junction proteins in brain capillary ECs in patients with active lesions, inactive lesions, as well as NAWM associated with fibrinogen leakage (Kirk et al., 2003; McQuaid et al., 2009; Bennett et al., 2010). More specifically, dysregulation of tight junction adaptor protein ZO-1, occludin and claudin-5 have been reported in both primary progressive and secondary progressive disease states (Kirk et al., 2003; Leech et al., 2007). Experimental autoimmune encephalomyelitis (EAE) in rodents is a disease model with clinical and pathological characteristics relevant to the study of MS. This model revealed reorganization of ZO-1 and actin in the presence of inflammatory factors *in vitro*, associated with increased permeability of an endothelial monolayer (Bennett et al., 2010). The EAE model also revealed increased expression of VEGF in ECs, astrocytes, monocytes and activated TH1 lymphocytes, all of which contribute to BBB permeability during the early phase of disease, while decreased expression VEGF was evident in the late phase (Girolamo et al., 2014). The increase in VEGF expression was also found in the brain of MS patients (Girolamo et al., 2014). Furthermore, junctional adhesion molecule-A, a component of tight junctions, was found abnormally distributed in active and inactive MS lesions, although adherent junction proteins were normally expressed and localized in MS tissue (Padden et al., 2007). In addition, levels of PECAM-1 were found increased in active gadolinium-enhancing MS lesions (Ortiz et al., 2014). While BBB leakage is evident in MS, the complex network of cellular and molecular players that lead to this dysfunction have yet to be fully understood. Targeting BBB defects in MS represent a therapeutic opportunity, for instance with MMP inhibitors, interferons, and corticosteroids (Minn et al., 2002; Ross et al., 2004; Pardridge, 2012; Ortiz et al., 2014). However, no current therapy addresses BBB deficits (Ortiz et al., 2014). For more details on BBB dysfunction in MS, the following reviews can be consulted (Girolamo et al., 2014; Kamphuis et al., 2015; Xiao et al., 2020).

ECs proliferation as well as an increase in vascular network density has been reported (Ludwin, 2006; Holley et al., 2010). Increased angiogenesis was suggested to contribute to disease progression as well as remission after relapses (Papadaki et al., 2014). In addition to increased VEGF levels, VEGFR2 is also expressed on ECs in active MS lesions (Seabrook et al., 2010). Other molecules, such as basic fibroblast growth factor, were increased in MS patients and involved in angiogenesis (Su et al., 2006). MS patients with activated lesions and NAWM show blood vessels with a glomeruloid morphology, hemorrhages and vessel wall hyalinization (Girolamo et al., 2014). Immunosuppressive therapies have been used in aggressive MS as they not only impact neuroinflammation but also have an anti-angiogenic effect. Further research is warranted to elucidate the vascular links to MS and identify new therapeutic targets, as disease modifying drugs have unfortunately little to no impact on MS progression (Girolamo et al., 2014).

### Vascular Links to Huntington’s Disease

HD is an hereditary, autosomal dominant and neurodegenerative disorder (Davenport, 1915; Wasmuth et al., 1988; Bano et al., 2011) leading to altered muscle coordination and declined mental abilities (Paulsen, 2011; Ha and Fung, 2012). An expansion of trinucleotide CAG repeats on chromosome 4 within the Huntingtin gene (*HTT)* results in the production of an altered Huntingtin (Htt) protein which accumulates in specific brain regions. Aggregation of mutant Htt (mHtt) leads to increased neurotoxicity (Zheng and Diamond, 2012), particularly in subcortical brain structures such as the neostriatum (caudate and putamen) where GABAergic medium-spiny neurons are particularly vulnerable (Sieradzan and Mann, 2001; Walker, 2007; Ross and Tabrizi, 2011; Drouin-Ouellet et al., 2015; McColgan and Tabrizi, 2018). At the cellular level, mHtt results in neuronal dysfunction and death through disrupted mechanisms involved in proteostasis, transcription and mitochondrial function as well as toxicity from the mutant protein (McColgan and Tabrizi, 2018). Worldwide, 2.71 per 100,000 individuals suffer from HD (Rawlins et al., 2016; Kounidas et al., 2021). Both men and women are affected equally, and heterogeneous symptoms emerge at around 40 years of age. However, functional and structural brain alterations emerge a decade before symptoms manifest (Snowden, 2017). Carriers of CAG repeat expansions in *HTT* can be identified decades before clinical manifestation, allowing researchers to identify possible biomarkers in the premanifest stage of HD (preHD). With this comes the increasing interest to study cerebrovascular abnormalities in HD (Snowden, 2017).

#### Altered Cerebral Blood Flow in Huntington’s Disease

HD-related perfusion deficits have been mostly associated with cerebral hypoperfusion (Reid et al., 1988; Sotrel et al., 1991; Hasselbalch et al., 1992; Harris et al., 1999; Deckel and Duffy, 2000; Wild and Fox, 2009). There is evidence of reduced CBF in the basal ganglia in early HD, prior to gross structural changes and to motor symptoms. In these cases, severity of cortical hypoperfusion correlated with decreased functional capabilities (Sax et al., 1996; Harris et al., 1999). In preHD patients, classified as either near or far from motor symptom onset, displayed altered rCBF by MR-based perfusion imaging. Participants with preHDfar and preHDnear had lower rCBF in the medial prefrontal cortex and increased rCBF in the left precuneus. Of note, structure and function of the precuneus and hippocampus can be abnormal in very early HD (Feigin et al., 2006). PreHDnear participants had additional regions showing altered rCBF, including hypoperfusion in the medial and lateral prefrontal cortex and hyperperfusion in the right hippocampus (Wolf et al., 2011).

While resting CBF is affected, early manifest and premanifest HD patients also display altered neurovascular coupling during visual stimulation (Klinkmueller et al., 2021). After HD onset, a significant hypoperfusion in the HD group was identified in most of the cerebral cortex. During problem-solving activities, such as solving a maze or resting their eyes open while looking at a modified maze, patients with HD showed increased CBF in the caudate nucleus (Deckel and Duffy, 2000; Deckel et al., 2000). Following physical activity, HD patients were associated with CBF hyperperfusion compared to the control group (Steventon et al., 2020).

Animal models of HD (e.g., gene knock-in of a human exon 1 CAG_140_ expansion repeat) also revealed altered rCBF. In mice as in humans, different brain regions displayed either hypoperfusion (basal ganglia motor circuit, hippocampus and prefrontal area) or hyperperfusion (cerebellar-thalamic and somatosensory regions). This altered CBF was apparent at a presymptomatic stage (Wang et al., 2016).

While CBF is starting to emerge as a biomarker for HD, mounting evidence supports the utilization of CBV as an additional metric. Several studies have reported elevated CBV in preHD patients (Hua et al., 2014; Liu et al., 2020). In addition, there is evidence of increased CBV in cortical gray matter after HD onset (Drouin-Ouellet et al., 2015), suggesting that arteriolar CBV may be a sensitive biomarker for premanifest HD (Hua et al., 2014; Liu et al., 2020). From these studies it was suggest that imaging of CBF may be used to detect widespread functional abnormalities in HD, and possibly predict HD symptoms onset during premanifest stages.

#### Altered Blood-Brain Barrier and Angiogenesis in Huntington’s Disease

Increases in vessel density, BBB permeability and VEGF-A release were observed in HD patients and animal models of HD (Steventon et al., 2020). There is evidence that BBB leakage increases alongside disease progression (Drouin-Ouellet et al., 2015). Despite these observations, there seems to be discrepancies between mouse models of HD. For instance, the BACHD transgenic mice, a well-known model of HD expressing the full-length mutant human *HTT*, failed to develop BBB breakdown at 12 months of age despite robust motor deficits (Lin et al., 2013; Mantovani et al., 2016). BBB dysfunction in HD patients has been associated with decreased tight junction molecules such as occludin and claudin-5 (Drouin-Ouellet et al., 2015). Moreover, other markers associated with BBB permeability, including hepatocyte growth factor, interleukin-8 and tissue inhibitor of MMP-1, were found elevated in HD patients (Drouin-Ouellet et al., 2015). A transgenic mouse model of HD (R6/2 mice) confirmed elevated tight junction molecules similar to HD patients. The R6/2 mouse model of HD is the most commonly studied and harbors a mutant *Htt* with CAG repeat expansion in exon 1 (Li et al., 2005). R6/2 mice also displayed increased transcytosis and paracellular transport across the brain endothelium compared to control mice (Drouin-Ouellet et al., 2015). In R6/2 mice, tight junction imbalance and perturbed BBB homeostasis were perceptible at very early stage of the disease, in absence of symptoms (Di Pardo et al., 2017). At the structural level, mHtt aggregates were found in the basal membrane of cerebral blood vessels in HD patients (Drouin-Ouellet et al., 2015). Interestingly, mHtt aggregates were localized in ECs, smooth muscle cells and perivascular macrophages, consistent with observations in R6/2 mice.

Further research is needed to determine BBB impairments in preHD patients. Lim et al. (2017) reported that iPSCs-derived brain microvascular endothelial cells (BMECs) from HD patients exhibit increased angiogenesis and altered barrier properties associated with elevated transcytosis and paracellular permeability. An increased and unregulated angiogenic activity may lead blood vessels to become more permeable with a potential role in neurovascular dysfunction in HD. RNA-seq analysis revealed a significant number of affected gene that regulate both clathrin- and caveolin- mediated endocytosis, which could lead to changes in endo- and transcytosis across the brain endothelium. These genes include *FABP4*, *DYNAMIN*, and *FILAMIN* that play a role in vesicle formation and scission. In addition, higher levels of transcytosis-related genes such as *CAV1* was detected in HD iPSCs-derived BMECs that also displayed impaired Wnt/β-catenin signaling (Lim et al., 2017). The Wnt/β-catenin pathway is essential for regulation of cell proliferation, cell determination and tissue homeostasis (Silva-Garcia et al., 2019). Furthermore, astrocytes from both HD patients and mouse models were associated with higher levels of VEGF-A, which may trigger proliferation of ECs and contributes to neurovascular changes in HD (Hsiao et al., 2015). Of note, sustained delivery of VEGF into the rat striatum via injectable hydrogels was neuroprotective in a lesioned model of HD; VEGF implants significantly protected against the quinolinic acid-induced loss of striatal neurons (Emerich et al., 2010). Moreover, neuroprotection induced by inhibition of hypoxia inducible factor (HIF) prolyl-4-hydroxylases in HD mice has been correlated with enhanced VEGF expression (Niatsetskaya et al., 2010). In post-mortem tissue, cerebral blood vessel density was greater in HD patients while no differences in diameter of small- or medium sized blood vessels have been observed (Drouin-Ouellet et al., 2015). Post-mortem tissue of HD patients revealed a higher proportion of small compared to medium-sized blood vessels in the putamen, an effect occurring in parallel with putamen degeneration. Notably, altered density of small blood vessels in HD patients was consistent with the R6/2 mouse model when brain vascular anomalies were restricted to smaller vessels (Drouin-Ouellet et al., 2015; St-Amour et al., 2015).

### Vascular Links to Parkinson’s Disease

PD is the second most common neurodegenerative disorder after AD (Antony et al., 2013). It is characterized by the progressive degeneration of the nigrostriatal system, resulting in rigidity, bradykinesia, postural instability, and resting tremor (Antony et al., 2013; Pagano et al., 2016). The most affected cells are dopaminergic neurons from the substantia nigra pars compacta (SNc). The pathological hallmark of PD is the formation of Lewy bodies containing aggregated α-synuclein (Hijaz and Volpicelli-Daley, 2020). While increasing age is a risk factor for PD, the average age of onset is after 60 years old (Hindle, 2010; Parkinson Canada, 2010). The etiology of PD is multifactorial where genetics (familial PD) and environmental (sporadic PD) factors take part in disease onset (Klein and Westenberger, 2012). Familial PD accounts for 10–15% of all PD cases whereas the remainder is classified as sporadic PD (Verstraeten et al., 2015). Genetically linked PD is inherited in an autosomal dominant or recessive fashion (Ball et al., 2019). Research has identified seven causal genes for familial PD including phosphatase and tensing homolog-induced Kinase-1 (PINK1), Parkinson protein 7 (PARK7), parkin RBR E3 ubiquitin protein ligase (PARK2), vacuolar protein sorting-associated protein 35 (VPS35), alpha-synuclein (SNCA), glucocerebrosidase (GBA) and leucine-rich repeat Kinase 2 (LRRK2) (Verstraeten et al., 2015; Kalinderi et al., 2016; Ball et al., 2019). Conversely, sporadic PD may develop from gene-environment interactions (Benmoyal-Segal and Soreq, 2006). Environmental factors associated with PD includes but are not limited to pesticides, heavy metals, and illicit drugs (Kwakye et al., 2017). Notably, individuals may respond differently to environmental factors which results in diverse symptomology of PD, thus adding to the complexity of the disease (Ball et al., 2019).

#### Altered Cerebral Blood Flow in Parkinson’s Disease

Using non-invasive MRI in an heterogeneous PD patient population, studies revealed decreased CBF in the frontal, parietal and occipital areas, more specifically the posterior parieto-occipital cortex, cuneus, middle frontal gyri, putamen, anterior cingulate and post- and pre-central gyri (Kamagata et al., 2011; Melzer et al., 2011; Fernandez-Seara et al., 2012; Madhyastha et al., 2015). A study by Fernandez-Seara et al. (2012) reported a 20–40% decrease in CBF in PD patients compared to a control group. Studies are trying to determine if CBF changes are related to the presence of dementia in PD, or if it can be considered as a biomarker. Derejko et al. (2006) used SPECT in PD patients with dementia and demonstrated left temporo-parietal hypoperfusion compared to the group without dementia. This suggested that CBF differences between PD patients with or without dementia could represent a clinical biomarker for discriminating PD patients (Derejko et al., 2006). Another study revealed hypoperfusion in PD patients without dementia in posterior cortical regions (posterior cingulate/precuneus) compared to healthy individuals (Syrimi et al., 2017). Hypoperfusion was positively correlated with global cognitive performance and the level of motor impairment (Madhyastha et al., 2015; Syrimi et al., 2017). Melzer et al. (2011) and Fernandez-Seara et al. (2012) reported CBF reduction with parietal cortex thinning in mild PD patients without dementia and proposed that CBF alterations occur in the early stages of PD.

Although studies have identified hypoperfusion in PD patients, the mechanisms underlying these changes are unknown (Biju et al., 2020). One study used a mouse model of PD (α-synuclein transgenic mice), which overexpress human WT α-synuclein. α-synuclein pathology develops before clinical symptoms and is present in both sporadic and familial forms. Using ASL-MRI analysis in this PD mouse model, authors reported a 36.6% reduction in cortical CBF in mutant mice accompanied by motor coordination impairments and olfactory bulb atrophy/dysfunction (Biju et al., 2020).

#### Altered Blood-Brain Barrier and Angiogenesis in Parkinson’s Disease

The association of PD with altered vascular function has led studies to investigate possible players contributing to BBB (Al-Bachari et al., 2020). In animal studies, BBB disruption in the SNc has been reported (Barcia et al., 2005; Rite et al., 2007; Chao et al., 2009). While human studies investigating BBB in PD patients are sparse, there is evidence of BBB dysfunction with increased permeability in the post commissural putamen of PD patients (Kortekaas et al., 2005; Gray and Woulfe, 2015). Wardlaw et al. (2008) and Al-Bachari et al. (2020) revealed increased leakage of the BBB in PD using ASL and dynamic contrast enhanced -MRI (DCE-MRI). Authors compared PD patients with two other control groups: one with and one without known cerebrovascular disease. This comparison could determine if BBB changes are attributable to co-existing cerebrovascular disease in an aging population, or if a pattern of BBB alteration is specific to PD. Authors reported increased BBB leakage in the group with cerebrovascular disease compared to the group without cerebrovascular disease in regions previously associated with PD, including the substantia nigra, white matter, and posterior cortical regions (Al-Bachari et al., 2020).

Accumulation of α-synuclein in ECs may also contribute to BBB dysfunction and increased permeability (Elabi et al., 2021). Higher number of EC nuclei was found in the SNc of PD patients (Faucheux et al., 1999). Other EC dysfunctions were reported, such as down regulation of tight junction proteins (Kuan et al., 2016). In the 1-methyl-4-phenyl-1,2,3,6-tetrahydropyridine (MPTP) mouse model of PD, down-regulation of tight junction protein ZO-1 and BBB leakage were measured in the substantia nigra (Patel et al., 2011). There is also evidence of string vessel formation in brain capillaries from human PD. String vessels are described as collapsed basement membrane without endothelium and no circulatory function. An altered basement membrane was also observed in PD mice (Yang et al., 2015). VEGF, a prominent growth factor promoting angiogenesis and BBB permeability, was upregulated in the substantia nigra, but not the striatum, of PD patients, while animal models of PD displayed parkinsonian traits following administration of exogenous VEGF into the substantia nigra (Barcia et al., 2005; Wada et al., 2006; Rite et al., 2007).

Guan et al. (2013) reported vascular degeneration in human PD, with formation of endothelial clusters, capillary network damage, and loss of capillary connections in the substantia nigra and brain stem nuclei. Authors found a larger vessel size in PD patients, while capillaries were shorter in average length, less in number and had fewer branches. These observations were also confirmed in an MPTP mouse model of PD (Guan et al., 2013; Sarkar et al., 2014). Furthermore, ultrastructural abnormalities were identified in cerebro-cortical microvessels of PD patients, including basement membrane thickening, vacuolization and pericyte degradation (Farkas et al., 2000). Structural alterations of the basement membrane can lead to pathophysiological consequences including compromised nutrient transport and cognitive disturbances (Farkas et al., 2000). Recently, a PD mouse model of α-synuclein overexpression was associated with altered vascular density at different stages of the disease (Elabi et al., 2021). The study reported that 8 month-old animals had increased vessel density compared to control mice, while 13 month-old PD mice displayed decreased vessel density, suggesting compensatory angiogenesis in the younger group (Elabi et al., 2021). Increased angiogenesis is considered an adaptative response to pathological conditions and is regulated by basement membrane proteins and their integrin receptors. These studies postulate that immature nascent vessels in PD could contribute to increased BBB permeability, as reviewed recently (Bogale et al., 2021).

### Vascular Links to Alzheimer’s Disease

AD accounts for 60–80% of all diagnoses of dementia (Alzheimer’s Association, 2021). This progressive and debilitating neurodegenerative disease manifests with memory, attention, executive, visuospatial and perceptual impairments. AD is not only characterized by amyloid deposition, neuroinflammation, neurodegeneration and cognitive deficits, but also by cerebrovascular pathology. Indeed, an inadequate brain perfusion has been identified as an early event in the development and progression of AD (Nicolakakis and Hamel, 2011). The risk of developing AD is increased by age-associated vascular diseases such as hypercholesterolemia, hypertension, ischemic stroke, and diabetes (Kalaria, 1996; Roher et al., 2003; Casserly and Topol, 2004; Gorelick, 2004; Luchsinger et al., 2005). The AD brain is characterized by increased levels of soluble and insoluble amyloid-beta peptide (Aβ), derived from the amyloid protein precursor (APP), neurofibrillary tangles of hyperphosphorylated *tau* protein, neurodegeneration and neuroinflammation, and also linked with a cerebrovascular pathology (Selkoe, 2002; Iadecola, 2004; Querfurth and LaFerla, 2010). The latter is identified post-mortem by Aβ deposition in brain vessels (cerebral amyloid angiopathy, CAA), Aβ-induced oxidative stress, and alterations of the vessel wall that included fibrosis and degeneration of ECs (Buee et al., 1994; Vinters et al., 1994; Zarow et al., 1997; Farkas and Luiten, 2001; Humpel and Marksteiner, 2005). Various mouse models of AD have been developed, most mimicking the overproduction of Aβ through transgene expression of mutated human APP (hAPP) combined or not with the amyloidogenic presenilin (PS1) or the pathologic *tau* (Mucke et al., 2000; Oddo et al., 2003; Gotz and Ittner, 2008). These models recapitulate AD’s cerebrovascular pathology in addition to the cognitive deficits, senile plaques, Aβ-induced oxidative stress, neuroinflammation, cholinergic denervation, synaptic failure, and cerebral hypometabolism (Hsia et al., 1999; Palop et al., 2003; Aucoin et al., 2005; Tong et al., 2005; Nicolakakis et al., 2008; Iturria-Medina et al., 2016; Love and Miners, 2016; Liu et al., 2018; Czako et al., 2020). It is in fact estimated that up to 45% of all dementias worldwide are partly, or wholly, due to age-related SVD of the brain (Montagne et al., 2016; Clancy et al., 2021). This suggests that AD and vascular dementia share common grounds, which complicates their stratification. As such, it is of utmost importance to improve our understanding of vascular underpinnings of AD (Willis and Hakim, 2013). Clinical studies that attempted to reduce plaque load by blocking Aβ production, removing Aβ with antibodies, or preventing tau phosphorylation, have all failed to alleviate AD symptoms (Korte et al., 2020). However, mounting evidence demonstrates that the brain vasculature is the missing link (Sweeney et al., 2019). Early cerebrovascular dysfunction in AD leads to decreased Aβ clearance, vascular oxidative stress, inflammatory damage and impaired BBB function (Zlokovic, 2005). Here below we will succinctly describe vascular underpinnings of AD, from alterations in CBF to BBB dysfunction, topics that have been extensively reviewed elsewhere (Bell and Zlokovic, 2009; Zlokovic, 2011; Hamel, 2015; Hays et al., 2016; Nelson et al., 2016; Kisler et al., 2017; Korte et al., 2020; Solis et al., 2020; Soto-Rojas et al., 2021).

#### Cerebral Blood Flow Alterations in Alzheimer’s Disease

Numerous investigations on individuals diagnosed with AD observed reduced CBF (Prohovnik et al., 1988; Montaldi et al., 1990; Bressi et al., 1992; O’Brien et al., 1992; Smith et al., 1992; Minoshima et al., 1997; Mattsson et al., 2014; Mielke et al., 2014; Smith and Verkman, 2018). CBF decline can be detected prior to cognitive decline, but also before plaque deposition. The accumulation of soluble Aβ prior to plaque deposition has early pathogenic consequences in AD (Suo et al., 1998). Studies have demonstrated increased levels of soluble amyloid species including Aβ_1__–__4__0_ and Aβ_1__–__4__2_ in AD cases compared to age-matched controls (Suo et al., 1998; Smith and Greenberg, 2009). Both soluble Aβ_1__–__4__0_ and Aβ_1__–__4__2_ have been associated to abnormal vascular reactivity in the absence of plaque deposition or vessel wall dysfunction (Smith and Greenberg, 2009; Dietrich et al., 2010). In particular, studies have revealed that application of exogenous Aβ_1__–__4__0_ to mouse neocortex *in vivo*, or to healthy bovine blood vessels *ex vivo*, leads to endothelium-dependent vasoconstriction (Thomas et al., 1996; Niwa et al., 2000). In addition, increased levels of soluble amyloid species (Aβ_1__–__4__0_ and Aβ_1__–__4__2_) are associated with significantly reduced CBF, increased cerebral vascular resistance, decrease myogenic and vasodilator responses (Suo et al., 1998; Dietrich et al., 2010), where Aβ_1__–__4__2_ is equally potent to Aβ_1__–__4__0_ except at a higher concentration (Dietrich et al., 2010). Soluble Aβ impacts vascular function through increased production of reactive oxygen species (ROS). The reaction of ROS superoxide and excess NO produces peroxynitrite. Peroxynitrite is commonly known as a toxic oxidant which contributes to endothelial dysfunction, a mechanism relevant to AD but also to other neuroinflammatory and metabolic conditions (Beckman et al., 1990; Paris et al., 1998; Tan et al., 2004; Dietrich et al., 2010; Kelleher and Soiza, 2013; Salisbury and Bronas, 2015; Incalza et al., 2018). Both Aβ_1__–__4__0_ and Aβ_1__–__4__2_ have been shown to acutely increase ROS production in cultured rat cerebral microvascular endothelial and smooth muscle cells in a dose dependent manner (Dietrich et al., 2010). Interestingly, this response was inhibited by the ROS scavenger MnTBAP (Dietrich et al., 2010). Notably, Aβ_1__–__4__0_ is the predominant isoform found in cerebral vessel walls and is commonly associated with vascular deposits in CAA, which will be discussed later, while Aβ_1__–__4__2_ is the major isoform deposited in senile plaques (Suo et al., 1998). Although this concept is still controversial, it is thought that Aβ_1__–__4__2_ acts as a “seed” which initiates the formation of vascular Aβ deposit in CAA (McGowan et al., 2005; Gireud-Goss et al., 2020).

Following Aβ deposition, reduction of CBF was found in the frontal, parietal and temporal cortices from individuals carrying Apolipoprotein E4 (*APOE4*) gene, most prevalent genetic risk factor for AD (Thambisetty et al., 2010; Michels et al., 2016). In addition, ApoE4 allele carriers displayed early impairments in cerebrovascular reactivity to a memory task (Suri et al., 2015). BOLD-fMRI, which uses blood flow changes as a surrogate to neuronal activity, detected decreased activation in areas engaged during naming and fluency tasks in AD patients compared to individuals with no risk factors (Smith et al., 1999). Decreased BOLD-fMRI responses to different cognitive tasks in early stage of AD are region-specific (Kisler et al., 2017). Most studies investigating perfusion in AD reported either CBF or CBV alterations. However, CBF alterations appear before CBV deficits during AD progression (Lacalle-Aurioles et al., 2014).

Decreased CBF is associated with poor cognitive function, and evidence suggested that lower CBF is linked with faster cognitive decline in patients with AD (Benedictus et al., 2017). Zheng et al. (2019) investigated rCBF, functional activity and connectivity in AD by combining resting-state BOLD fMRI and ASL techniques. ASL revealed decreased rCBF in AD patients in the left posterior cingulate cortex, bilateral dorsolateral prefrontal cortex, left interior parietal lobule, right middle temporal gyrus, left middle occipital gyrus and left precuneus. In addition, they revealed decreased connectivity between regions in AD patients, which was associated with impaired cognitive performances (Alsop et al., 2000; Zheng et al., 2019). Brain regions affected by a reduction of CBF in AD patients (parietal, frontal, temporal and occipital cortices) are associated with cognitive impairment in all domains (language, global cognition, memory, attention, executive functioning and visuospatial functioning) (Leeuwis et al., 2017).

Blood flow reductions have also been identified in early preclinical AD, before Aβ plaque deposition (Nicolakakis and Hamel, 2011; Iturria-Medina et al., 2016; Szu and Obenaus, 2021). Early reduction of CBF has been reported in mouse models of AD, such as mice overexpressing mutant forms of APP (Niwa et al., 2002; Ongali et al., 2010; Lacoste et al., 2013) and in mice expressing the *ApoE4* gene allele (Lin et al., 2017). In some brain areas, CBF reduction can reach over 50%. This CBF reduction has been associated with cognitive changes in mice, including a loss of ability to sustain attention (Marshall et al., 2001). Both *ApoE4* transgenic and APP/PS1 mice revealed CBF reduction prior to neuronal and synaptic dysfunctions (Guo et al., 2019; Montagne et al., 2021).

While decreased CBF in AD is widely accepted, studies are only starting to identify underlying mechanisms, for example the involvement of pericytes. Pericytes have been linked to hypoperfusion and increased capillary constriction in AD (Bell et al., 2010; Korte et al., 2020). Pericyte-deficient transgenic mice with no Aβ pathology develop early CBF reduction in the gray matter, even with normal neuronal activity, endothelial-dependent vasodilation, astrocyte number and blood vessels coverage (Bell et al., 2010; Kisler et al., 2017). As these pericyte-deficient mice age, neuronal dysfunction and degeneration start to emerge. Another underlying mechanism was reported by Cruz Hernandez et al. (2019), demonstrating that capillaries become blocked by neutrophils, while another study revealed increased formation of occlusive thrombi in AD mice (Cortes-Canteli et al., 2019). Inhibiting neutrophils adhesion using an antibody against neutrophil-specific protein Ly6G in the APP/PSI mouse model led to rapid improvements in CBF (Cruz Hernandez et al., 2019). In a follow-up study, the same group assessed the impact of one treatment of anti-Ly6G on short-term memory function and reported increased CBF by 17% in 21–22 months old APP/PSI mice. Furthermore, they suggested that increased CBF improved cognition into late stages of AD mice (Bracko et al., 2020). Reduced neurovascular coupling and cerebrovascular reactivity have also been reported in AD mice (Girouard and Iadecola, 2006; Tong et al., 2019). Recently, impaired capillary endothelial inward rectifying Kir2.1 channel, playing a role in mediating blood delivery, has been associated with AD (Mughal et al., 2021). In a model of familial AD (5xFAD) where Kir2.1 channel function is impaired, systemic administration of the co-factor phosphatidylinositol 4,5-bisphosphate (PIP_2_), required for Kir2.1 activity, led to increased CBF and functional neurovascular coupling in 5xFAD mice (Mughal et al., 2021).

AD patients are often (80–90%) diagnosed with CAA, a vessel disorder (Gireud-Goss et al., 2020) and an important risk factor for intracerebral hemorrhage and cognitive impairment (Reijmer et al., 2016). CAA consist of vascular amyloid deposits similar to senile plaques in AD (Kumar-Singh et al., 2005). Neuropathological studies have revealed that CAA affects the outer leptomeningeal vessels on the surface of the brain as well as distal intraparenchymal arteries, arterioles, and capillaries (Gireud-Goss et al., 2020; Howe et al., 2020). APP23 mouse model and human AD brain revealed an association between CAA-related capillary occlusion with CBF disturbances, hypoperfusion, detected by magnetic resonance angiopathy (MRA), which could explain in part the changes in CBF measured in AD patients (Thal et al., 2009; Milner et al., 2014). As in AD, patients with CAA have been linked to altered hemodynamics during visual stimulation as evidenced by reduced amplitude of BOLD response (Smith et al., 2008; Dumas et al., 2012; Switzer et al., 2020).

#### Altered Blood-Brain Barrier and Angiogenesis in Alzheimer’s Disease

Early signs of BBB leakage in AD have been detected before dementia onset (Montagne et al., 2016). Neuroimaging techniques have evidenced BBB breakdown in AD in gray and white matter brain regions (Montagne et al., 2016; van de Haar et al., 2016). Aβ and tau pathologies contribute to increased BBB permeability in AD patients and mouse models (Park et al., 2011; Sagare et al., 2013; Alata et al., 2015). Several players involved in Aβ clearance, and closely related to the BBB, are reduced in AD patients, including phosphatidylinositol-binding clathrin assembly protein (PICALM, allows for Aβ exocytosis across the luminal part of the BBB), P-glycoprotein (expressed on both sided of the BBB) and glucose transporter (GLUT)1 (Mooradian et al., 1997; Chiu et al., 2015; Zhao et al., 2015). AD brain microvessel show diminished expression of LRP1, a major Aβ clearance receptor at the BBB (Deane et al., 2004; Donahue et al., 2006). LRP1 is an ApoE receptor and is expressed at the abluminal side of brain ECs and mediates the internalization of soluble Aβ (Deane et al., 2004). Endothelium-specific deletion of LRP1 leads to the acceleration of Aβ pathology in APP-overexpressing APP_*sw/0*_ mice (Storck et al., 2016). Moreover, studies have demonstrated low levels of GLUT1 in AD brain endothelium, which alters glucose transport (Kalaria and Harik, 1989; Simpson et al., 1994).

Several features lead to increased BBB permeability in AD, including reduced expression of tight junctions, perivascular accumulation of blood-derived products, degeneration of pericytes and ECs, as well as infiltration of circulating leukocytes (Sweeney et al., 2018; Huang et al., 2020). It was demonstrated that Aβ disrupts tight junctions and increases vascular permeability by suppressing expression of ZO-1, claudin-5 and occludin while increasing expression of MMP-2 and MMP-9 (Kook et al., 2012; Blair et al., 2015; Wan et al., 2015; Huang et al., 2020). Isolated rat cerebral cortical ECs treated with Aβ_1__–__4__2_ displayed decreased expression of occludin and redistribution of claudin-5 and ZO-2 in the cytoplasm while in untreated cells, both claudin-5 and ZO-2 were distributed along the plasma membrane at cell-cell contacts (Marco and Skaper, 2006). In addition, studies have reported leakage of blood-derived proteins (fibrinogen, thrombin, albumin, and IgG) around capillaries from post-mortem brain tissue in the prefrontal and entorhinal cortex as well as in hippocampus of AD patients (Ryu and McLarnon, 2009; Hultman et al., 2013; Sengillo et al., 2013). Furthermore, animal studies revealed that lacking pericyte-derived soluble factors, required for a healthy endothelium, can contribute to endothelial degeneration in AD (Bell et al., 2010). Finally, mouse models of AD have demonstrated that pericyte reduction is associated with BBB dysfunction as well as accelerated buildup of Aβ and tau pathology (Sagare et al., 2013). In human studies, there is also evidence of pericyte loss in the hippocampus and cortex of AD patients due in part to prolonged exposure to Aβ peptides (Sagare et al., 2013; Sengillo et al., 2013; Huang et al., 2020). Of note, pericytes play a role in Aβ clearance by internalizing different Aβ peptides using the LRP1 pathway (Sagare et al., 2013).

Evidence of reduced capillary length and basement membrane changes in AD patients have been reported (Salloway et al., 2002; Sengillo et al., 2013; Halliday et al., 2016). It was shown that AD patients display abnormal angiogenesis due to low expression of MEOX2, a regulator of vascular differentiation, as well as premature pruning of capillary networks resulting in reductions of CBF (Wu et al., 2005; Grammas, 2011). Endothelial degeneration including reduction of EC thickness, length and density of blood vessels were reported in brain tissue from AD patients (Sweeney et al., 2018). An increase of pro-angiogenic factors in the AD brain, without the increase in vasculature, was also reported (Grammas, 2011). Notably, the increased Aβ species and plaques in AD have anti-angiogenic effects (Parodi-Rullan et al., 2020), and impaired angiogenesis was identified in transgenic AD mice (Grammas, 2011). Emerging evidence suggest that dysfunction of the VEGF-A/VEGFR2 pathway may play an aggravating role in neurodegeneration and AD. For instance, sustained brain delivery of VEGF via injectable hydrogels was protective against quinolinic acid-induced neurodegeneration (Emerich et al., 2010), and low VEGF levels have been associated to another debilitating neurological disorder, spinocerebellar ataxia type 1 (Cvetanovic et al., 2011). Aβ acts as an antagonist of VEGF signaling via sequestration of VEGF-A in senile plaques, and also via inhibition of VEGFR2 tyrosine phosphorylation (Patel et al., 2010). Moreover, implantation of VEGF secreting microcapsules on the cerebral cortex of APP/PS1 mice attenuated both brain Aβ burden and cognitive impairments (Spuch et al., 2010). Whether impaired neural perfusion and increased neurotoxicity in AD correlate to a loss of VEGF function, and whether VEGF overexpression is neuroprotective in transgenic AD mice remains to be explored.

CAA is associated with increased BBB permeability and arterial stiffness (Magaki et al., 2018; Gireud-Goss et al., 2020). Aβ deposition in CAA has been found to occur on the cerebrovascular basement membrane of arteries, arterioles and on the basal lamina of capillaries as shown by electron microscopy (Gireud-Goss et al., 2020). Moreover, ultrastructural studies of CAA demonstrated a thinned endothelium, shrinkage and degeneration of ECs, as well as vessel occlusion, all of which can lead to CBF disturbances and microinfarcts (Attems and Jellinger, 2004; Thal et al., 2009; Magaki et al., 2018). Tight junction proteins in CAA-laden vessels are found decreased (Tai et al., 2010). After exposure to exogenous Aβ, human ECs showed decreased expression of occludin, while post-mortem brain tissue of CAA patients revealed decreased expression of claudin-5, ZO-1, CD31 and basement protein collagen IV (Tai et al., 2010; Carrano et al., 2011; Magaki et al., 2018). In addition, CAA patients displayed increased expression of MMP-2 and MMP-9, which may lead to basement membrane degradation and increased BBB permeability (Carrano et al., 2011). In the Tg2576 mouse model of CAA, BBB integrity was compromised due to decreased expression of claudin-5 and claudin-1 (Carrano et al., 2011). Moreover, TgSwDI mice, another model of CAA, revealed spontaneous hemorrhage and loss of BBB integrity (Davis et al., 2004). Soluble Aβ_1__–__4__0_, predominant amyloid isoform in vessel walls, also leads to tight junction redistribution at the BBB and decreased transendothelial electric resistance (Hartz et al., 2012; Gireud-Goss et al., 2020). Understanding the impact of Aβ in CAA and AD is essential for slowing cerebrovascular disease progression.

## Additional Remarks: Vascular Deficits in Down Syndrome, Traumatic Brain Injury and Depression

In addition to neurodevelopmental disorders discussed earlier in this review, Down syndrome (DS), which results from trisomy of human chromosome 21, is a cause of early onset Alzheimer’s disease-dementia (AD-DS) (Ballard et al., 2016; Tosh et al., 2021). Two-thirds of individuals with DS will develop dementia by the age of 65 (Tosh et al., 2021). The onset of AD in DS patients parallels the development of the classic brain pathological lesions seen in AD patients without DS (Salehi et al., 2016). DS and AD disorders have genetic similarities, as individuals with DS possess a triplication of the gene encoding APP, while patients with familial AD have an extra copy of the APP gene (Salehi et al., 2016). In rodent studies of DS-AD, triplication of chromosome 21 genes other than *APP* demonstrated increased Aβ aggregation deposition and cognitive deficits (Wiseman et al., 2018). A recent study, focused on a model of DS comprising of a mutation in a Down syndrome critical region (Hsa21) on chromosome 21 encompassing 21q21–21q22.3 (Li et al., 2016; Tosh et al., 2021). This study crossed an Hsa21 mouse model of DS with partial trisomies other than *APP* with a transgenic APP mouse model and revealed that an additional copy of genes of the Hsa21 region modulates APP/Aβ biology, including Aβ aggregation and mortality (Tosh et al., 2021). Despite striking similarities between AD and DS in terms of genetics and symptoms onset, neurovascular impairments in DS have been largely overlooked. As such, studies aimed at elucidating vascular abnormalities in DS represent an unmet clinical need.

Early vascular insults following a traumatic brain injury (TBI) can also increase the risk of late-onset neurological diseases (Brett et al., 2021). TBI is a significant public health problem associated with long-term disabilities. Early chronic TBI may lead to secondary injury with pathophysiological changes similar to those observed in neurodegenerative diseases (Impellizzeri et al., 2016). For instance, neuroinflammation plays a fundamental role in TBI, including reactive microglia and astrocytes, as well as release of pro-inflammatory cytokines and chemokines that may hinder the brain’s ability to repair itself and lead to neurodegeneration following prolonged activation of these processes (Impellizzeri et al., 2016; Brett et al., 2021). Severe or repeated mild TBI can initiate long-term neurodegeneration with signs of AD (Mendez, 2017). For example, various contact-sport players developed TBI-associated dementia or parkinsonism years after retiring. TBI can induce acute BBB disruption through vascular shear stress, hemorrhages, edema, alterations in CBF and chronic inflammation, which is known to contribute to Aβ deposition and tau pathology (Iadecola, 2013; De Silva and Faraci, 2016). Autopsies of TBI patients show diffuse Aβ plaques similar to those identified in AD, as reviewed by Perry et al. (2016). The formation of Aβ in perivascular spaces following TBI may lead to an injury cascade consisting of cerebrovascular damage, oxidative stress and ECs dysfunction (Ramos-Cejudo et al., 2018). Interestingly, alterations in EC survival, BBB integrity and neuroinflammation are considered early events after TBI, all of which are characteristic of cerebrovascular damage involved in the progression of AD and impairment of Aβ clearance. Thus, these early vascular impairments promote the onset of neurodegenerative diseases (Ramos-Cejudo et al., 2018). Considering early vascular injuries in TBI, biomarker studies are integrating a variety of neuroimaging and molecular techniques to better understand the incidence of cerebrovascular dysfunction and the onset of neurodegenerative diseases, and therapeutic investigations have looked at ways to improve cerebrovascular function (Graham and Sharp, 2019; Martinez and Stabenfeldt, 2019).

One of the leading causes of mental illness worldwide, depression, has a tremendous impact on psychosocial behaviors and vascular health (Knight and Baune, 2017; Menard et al., 2017). Chronic stress is the primary environmental risk factor for depression. The nucleus accumbens (NAc) is one of the main players in regulating stress response (Russo and Nestler, 2013). Menard et al. (2017) have demonstrated that chronic social stress induces BBB leakiness in the NAc of mice, which leads to circulating proinflammatory mediators and depression-like behaviors such as helplessness, social avoidance and anhedonia. As seen in neurodevelopmental and neurodegenerative disorders, the increase in BBB permeability in the rodent model of chronic social stress was facilitated by the loss of tight junction protein claudin-5 (Menard et al., 2017). Furthermore, stress-induced BBB permeability has been linked to inflammation of the endothelium and up-regulation of an epigenetic repressor, *hdac1*, which is involved in reducing claudin-5 expression and loosening of tight junctions (Dudek et al., 2020). Consequently, these studies are highlighting mechanisms by which chronic stress impacts vascular health, which could have long-term consequences on brain maturation and aging.

The vascular system, as any other system, undergoes aging. It has been hypothesized that vascular aging leads to a progressive functional deterioration (Grunewald et al., 2021). During aging, the brain vasculature undergoes several changes including decreased capillary density, attenuation of neovascularization potential, increased BBB permeability and decreased CBF as reviewed in Watanabe et al. (2020) and Banks et al. (2021). A suggested mechanism of typical vascular aging consist of the inability of VEGF to replenish vessel loss. The mechanisms by which VEGF is involved in vascular aging are unknown. However, mice treated with VEGF have been shown to live longer, with extended multiorgan functionality (Grunewald et al., 2021). Furthermore, aging is associated with several vascular changes including aortic stiffness which has been linked to reduced blood flow in tissues leading to increased neuroinflammation and neurodegeneration later in life (Moore et al., 2021). Therefore, age-related changes in key vascular features may predispose to age-associated diseases (Banks et al., 2021). Improving early pathological conditions by protecting the brain vasculature is essential in preventing or modulating disease progression.

## Conclusion

Vascular risk factors and co-morbidities take part in disease onset and/or exacerbate disease progression (Sweeney et al., 2018; Clancy et al., 2021). When it comes to alterations in CBF, BBB, and vascular patterning, neurodevelopmental and neurodegenerative disorders share interesting similarities (Table 1). While these disorders are siloed, mainly due to the age of onset, the commonalities in vascular alterations force to question the implication of early life vascular impairments on the expression of age-related neurodegenerative diseases. The vascular implications in middle-aged autistic adults have been largely overlooked, 10% of individuals diagnosed with ASD age between 40 and 60 years old will develop dementia, including AD within 15 years (Plana-Ripoll et al., 2019). In addition, there is a high frequency of parkinsonism among older ASD patients (Starkstein et al., 2015). The impact of altered brain perfusion and BBB integrity in ASD may contribute to the onset of neurodegenerative diseases due to the continuous vascular impairments associated with these diseases. Likewise, schizophrenia is associated with an elevated risk for developing Alzheimer’s and Parkinson’s diseases as they share core features including white matter abnormalities and cognitive deficits (Ribe et al., 2015; Kochunov et al., 2021; Kuusimaki et al., 2021).

**TABLE 1:** Major altered features associated with CBF, BBB, and angiogenesis in neurodevelopmental and neurodegenerative disorders.

Disorder	Key features	Selected references
**ASD**		
Altered CBF	– Widespread cerebral hypoperfusion in 75% of ASD children associated with language deficits, impaired executive function and abnormal response to sensory stimuli. – Hyperperfusion identified in frontotemporal regions. – Reduced hemodynamic responses. – Cerebral hypoperfusion also identified in rodent models of ASD. – Increased resting CBF and decreased NVC in an adult mouse model of ASD associated with endothelial dysfunction.	Ohnishi et al., 2000; Zilbovicius et al., 2000; Burroni et al., 2008; Reynell and Harris, 2013; Jann et al., 2015; Ouellette et al., 2020; Uratani et al., 2019

Altered BBB and angiogenesis	– Reduced level of adhesion molecules (CD31 and P-selectin). – Increased MMP-9 which regulates cell proliferation, adhesion, angiogenesis, oxidative injury and BBB breakdown. – Altered expression of claudin-5 and claudin-12. – Increased BBB permeability and impaired angiogenesis in animal models. – Reduced angiogenesis found in a mouse model of ASD.	Onore et al., 2012; Kumar et al., 2015; Azmitia et al., 2016; Fiorentino et al., 2016; Turner and Sharp, 2016; Ouellette et al., 2020

**Schizophrenia**		
Altered CBF	– Increased CBF in the cingulate gyrus and superior frontal gyrus associated with positive symptoms. – Negative symptoms associated with hypoperfusion in the superior temporal gyrus bilaterally and left middle frontal gyrus. – rCBF alterations depend on severity of positive symptoms. – Increased CBF in the right superior temporal gyrus and caudate nucleus. – Decreased CBF in the occipital and left parietal cortices. – Altered NVC including reduced amplitude of response and delayed hemodynamics.	Sabri et al., 1997; Carter et al., 2001; Schultz et al., 2002; Malaspina et al., 2004; Ford et al., 2005; Pinkham et al., 2011; Liu et al., 2012; Kawakami et al., 2014; Pu et al., 2016; Zhuo et al., 2017

Altered BBB and angiogenesis	– Increased BBB permeability. – Thickening and deformation of basal lamina, vacuolation of EC cytoplasm, swelling of astrocyte end-feet, activation of microglial cells and atypical vascular arborization in prefrontal and visual cortices. – Decreased claudin-5 expression, altered level of VE-cadherin and occludin in ECs. – Impaired angiogenesis and VEGF upregulation in the prefrontal cortex linked to vascular hyperpermeability.	Grove et al., 2015; Hino et al., 2016; Casas et al., 2018; Carrier et al., 2020; Cai et al., 2020; Guo et al., 2020; Crockett et al., 2021; Usta et al., 2021

**MS**		
Altered CBF	– Hypoperfusion in SP-MS, RR-MS and PP-MS patients. – Active demyelinating lesions associated with hyperperfusion and stable lesions linked to hypoperfusion. – CBF alterations present in early stages of disease. – Impaired cerebral vascular reactivity leads to neuronal death. – Overproduction of NO desensitize EC and smooth muscle cell function, leading to decreased vasodilatory capacity and limited blood supply to neurons.	Ge et al., 2005; Varga et al., 2009; D’Haeseleer et al., 2011; Ota et al., 2013; Marshall et al., 2014; Bester et al., 2015; Monti et al., 2018; Hostenbach et al., 2019

Altered BBB and angiogenesis	– BBB hyperpermeability. – Decreased expression of TJ proteins (ZO-1, occludin and claudin-5) in ECs in patients with active and inactive lesions. – Rodent model of MS show increased expression of VEGF in ECs, astrocytes and monocytes. – Increased vascular network density and angiogenesis.	Kirk et al., 2003; Bennett et al., 2010; Holley et al., 2010; Cramer et al., 2014; Girolamo et al., 2014; Papadaki et al., 2014

**HD**		
Altered CBF	– Altered CBF prior to structural changes and motor symptoms. – Cerebral hypoperfusion in the basal ganglia, medial and lateral prefrontal cortex. – Cerebral hyperperfusion in the cerebellar-thalamic and somatosensory regions. – Altered neurovascular coupling during visual stimulation.	Hasselbalch et al., 1992; Sax et al., 1996; Deckel and Duffy, 2000; Wang et al., 2016; Klinkmueller et al., 2021
Altered BBB and angiogenesis	– Increased vessel density, BBB leakage and VEGF-A release. – Decreased TJ molecules including occludin and claudin-5. – Rodent model of HD revealed increased transcytosis and paracellular transport in brain ECs with TJ imbalance. – mHtt aggregates localized in ECs, smooth muscle cells and perivascular macrophages. – iPSCs-derived HD BMECs show increased angiogenesis, altered barrier properties and impaired Wnt/β-catenin signaling.	Steventon et al., 2020; Drouin-Ouellet et al., 2015; Di Pardo et al., 2017; Lim et al., 2017
**PD**		
Altered CBF	– Decreased CBF in frontal, parietal and occipital areas. – PD patients with dementia show left temporo-parietal hypoperfusion. – PD patients without dementia display hypoperfusion in the posterior cortical regions. – Hypoperfusion is positively correlated with cognitive performance and motor impairment.	Derejko et al., 2006; Kamagata et al., 2011; Fernandez-Seara et al., 2012; Madhyastha et al., 2015; Syrimi et al., 2017

Altered BBB and angiogenesis	– BBB disruption in the SNc with increased permeability in the post-commissural putamen. – Down regulation of TJ proteins (ZO-1) and higher number of EC nuclei in the SNc. – String vessel formation in brain capillary networks. – Upregulation of VEGF, and parkinsonian traits following VEGF administration in rodent models. – Formation of endothelial clusters, capillary network damage, loss of capillary connections in the SN, basement membrane thickening, vacuolization, and pericyte degradation.	Farkas et al., 2000; Barcia et al., 2005; Kortekaas et al., 2005; Wada et al., 2006; Rite et al., 2007; Chao et al., 2009; Patel et al., 2011; Guan et al., 2013; Yang et al., 2015; Kuan et al., 2016

**AD**		
Altered CBF	– Reduced CBF prior to cognitive decline and plaque deposition. – Soluble Aβ_1__–__4__0_ and Aβ_1__–__4__2_ are associated with abnormal vascular reactivity and decreased myogenic responses in absence of plaque deposition. – Hypoperfusion detected following Aβ deposition in the frontal, parietal and temporal cortices and poor cognitive function. – BOLD-fMRI detected decreased activation in regions involved in naming and fluency tasks. – Hypoperfusion identified in rodent models overexpressing mutant forms of APP. – Rodent models show reduced NVC and cerebrovascular reactivity. – Parallel diagnosis of CAA linked with altered hemodynamics, capillary occlusion and hypoperfusion.	Montaldi et al., 1990; Bressi et al., 1992; Smith et al., 1999; Marshall et al., 2001; Girouard and Iadecola, 2006; Smith and Greenberg, 2009; Dietrich et al., 2010; Ongali et al., 2010; Dumas et al., 2012; Lacoste et al., 2013; Mattsson et al., 2014; Milner et al., 2014; Benedictus et al., 2017; Smith and Verkman, 2018

Altered BBB and angiogenesis	– Aβ and tau pathologies contribute to BBB breakdown, reduced expression of TJ (ZO-1, claudin-5, occludin) and degeneration of pericytes and ECs. – Brain microvessel with diminished expression of LRP1. – Reduced level of GLUT1 in brain endothelium. – Reduced capillary length with basement membrane alterations. – Abnormal angiogenesis related to low expression of MEOX2. – Reduced EC thickness, and lower length/density of blood vessels. – Dysfunction of the VEGF-A/VEGFR2 pathway aggravates neurodegeneration. – Rodent models show pericyte loss. – Aβ deposition in CAA linked to decreased TJ proteins, increased expression of MMP-2 and MMP-9, thinned endothelium, degeneration of ECs and leaky BBB.	Kalaria and Harik, 1989; Simpson et al., 1994; Emerich et al., 2010; Tai et al., 2010; Grammas, 2011; Carrano et al., 2011; Sagare et al., 2013; Halliday et al., 2016; Montagne et al., 2016; van de Haar et al., 2016; Magaki et al., 2018; Sweeney et al., 2018; Huang et al., 2020

*Selected references are displayed. Aβ, β-amyloid peptide; AD, Alzheimer’s disease; APP, amyloid precursor protein; ASD, autism spectrum disorders; BBB, blood brain barrier; BMECs, brain microvascular endothelial cells; BOLD-FMRI, blood oxygen level dependent imaging-functional magnetic resonance imaging; CAA, cerebral amyloid angiopathy; CBF, cerebral blood flow; ECs, endothelial cells; GLUT1, glucose transporter 1; HD, Huntington’s disease; iPSC, induced pluripotent stem cells; LRP1, low-density lipoprotein receptor-related protein 1; MEOX2, Mesenchyme Homeobox 2; mHtt, mutant huntingtin; MMP, matrix metalloproteinases; MS, multiple sclerosis; NO, nitric oxide; NVC, neurovascular coupling; PD, Parkinson’s disease; PP-MS, primary progressive-multiple sclerosis; rCBF, regional cerebral blood flow; RR-MS, relapsing remitting-multiple sclerosis; SN, substantia nigra; SNc, substantia nigra pars compacta; SP-MS, secondary progressive-multiple sclerosis; TJ, tight junctions; VE-cadherin, vascular endothelial cadherin; VEGF, vascular endothelial growth factor; VEGFR2, vascular endothelial growth factor receptor 2; Wnt/β-catenin, Wingless-related integration site β- catenin; ZO-1, Zonula occludens-1.*

Since fast-growing evidence demonstrates the role of early vascular impairments in the onset and/or progression of numerous neurological conditions, more work is needed to identify therapeutic targets to promote healthy cerebrovascular maturation and aging, as well as hinder the progression of age-related dementia and neurodegeneration. This is primordial considering recent findings that ECs show limited turnover compared to other cells in the human body (Sender and Milo, 2021). For instance, it was estimated that the turnover rate of ECs is 0.1% per day, as opposed to much higher rates for erythrocytes (65%), neutrophils (18%) or gastrointestinal epithelial cells (12%). In addition, the turnover rates of cellular mass in the human body were estimated at 0.4% for ECs, 4% for skin cells and adipocytes, and 42% for gastrointestinal epithelial cells (Sender and Milo, 2021). Hence, as ECs are long-lived, they may carry on early structural and functional impairments into adulthood and throughout aging, altering organ function in the long term. This concept emphasizes the importance of infant screening for cerebrovascular abnormalities, and of continuous management of vascular risk factors during lifespan. As such, the vascular continuum between neurodevelopmental and neurodegenerative disease should represent a growing focus in modern neuroscience (Figure 3).

## Author Contributions

JO wrote the draft following BL’s instructions. BL chose the theme and edited the manuscript. Both authors contributed to the article and approved the submitted version.

## Conflict of Interest

The authors declare that the research was conducted in the absence of any commercial or financial relationships that could be construed as a potential conflict of interest.

## Publisher’s Note

All claims expressed in this article are solely those of the authors and do not necessarily represent those of their affiliated organizations, or those of the publisher, the editors and the reviewers. Any product that may be evaluated in this article, or claim that may be made by its manufacturer, is not guaranteed or endorsed by the publisher.
